# Mitochondrial co-chaperone protein Tid1 is required for energy homeostasis during skeletal myogenesis

**DOI:** 10.1186/s13287-016-0443-8

**Published:** 2016-12-07

**Authors:** Li-Hao Cheng, Kai-Feng Hung, Te-Chang Lee, Chih-Yang Huang, Wen-Ting Chiu, Jeng-Fan Lo, Tung-Fu Huang

**Affiliations:** 1 Institute of Oral Biology, National Yang-Ming University, Taipei, Taiwan, Republic of China; 2 Department of Dentistry, School of Dentistry, National Yang-Ming University, Taipei, Taiwan, Republic of China; 3 Institute of Biomedical Sciences, Academia Sinica, Taipei, Taiwan, Republic of China; 4 Graduate Institute of Chinese Medical Science and Institute of Medical Science, China Medical University, Taichung, Taiwan, Republic of China; 5 Institute of Basic Medical Science, China Medical University, Taichung, Taiwan, Republic of China; 6 Department of Health and Nutrition Biotechnology, Asia University, Taichung, Taiwan, Republic of China; 7 Department of Dentistry, Taipei Veterans General Hospital, Taipei, Taiwan, Republic of China; 8 Genome Research Center, National Yang-Ming University, Taipei, Taiwan, Republic of China; 9 National Yang-Ming University VGH Genome Research Center, Taipei, Taiwan, Republic of China; 10 School of Medicine, National Yang-Ming University, Taipei, Taiwan, Republic of China; 11 Department of Orthopedics and Traumatology, Taipei Veterans General Hospital, Taipei, Taiwan, Republic of China

**Keywords:** Skeletal muscle, Co-chaperone, ATP, AMPK and PGC-1α

## Abstract

**Background:**

Tid1 is a mitochondrial co-chaperone protein and its transcript is abundantly expressed in skeletal muscle tissues. However, the physiological function of Tid1 during skeletal myogenesis remains unclear.

**Methods:**

In vitro induced differentiation assay of mouse myoblast C2C12 cells was applied to examine the physiological role of Tid1 during skeletal myogenesis. In addition, transgenic mice with muscle specific (HSA-Cre) Tid1 deletion were established and examined to determine the physiological function of Tid1 during skeletal muscle development in vivo.

**Results:**

Expression of Tid1 protein was upregulated in the differentiated C2C12 cells, and the HSA-Tid1^f/f^ mice displayed muscular dystrophic phenotype. The expression of myosin heavy chain (MyHC), the protein served as the muscular development marker, was reduced in HSA-Tid1^f/f^ mice at postnatal day (P)5 and P8. The protein levels of ATP sensor (p-AMPK) and mitochondrial biogenesis protein (PGC-1α) were also significantly reduced in HSA-Tid1^f/f^ mice. Moreover, Tid1 deficiency induced apoptotic marker Caspase-3 in muscle tissues of HSA-Tid1^f/f^ mice. Consistent with the in vivo finding, we observed that downregulation of Tid1 not only reduced the ATP production but also abolished the differentiation ability of C2C12 cells by impairing the mitochondrial activity.

**Conclusion:**

Together, our results suggest that Tid1 deficiency reduces ATP production and abolishes mitochondrial activity, resulting in energy imbalance and promoting apoptosis of muscle cells during myogenesis. It will be of importance to understand the function of *Tid1* during human muscular dystrophy in the future.

**Electronic supplementary material:**

The online version of this article (doi:10.1186/s13287-016-0443-8) contains supplementary material, which is available to authorized users.

## Background

Muscles are soft tissues and function to produce force and motion in animals. Muscles are primarily responsible for posture and locomotion, as well as movement of internal organs such as the contraction of the heart and the movement of food through the digestive system [1]. Muscle tissues are derived from the mesodermal layer during embryogenesis in a process known as myogenesis [2]. Muscles are primarily powered by the oxidation of fats and carbohydrates, but anaerobic chemical reactions are also used, particularly by fast twitch fibers [3]. These catabolic reactions produce adenosine triphosphate (ATP) molecules which are used to power the contraction of muscle cells [3]. Dysfunction of muscles can cause muscle fatigue and severe diseases [4]. As reported by others, the AMPK signaling pathways are important to maintain the energy homeostasis of muscle tissues [5–7].

Accumulating studies suggest that mitochondria are associated with the regulation of the skeletal muscle physiology [8–15]. In addition, increase of mitochondrial biogenesis and mitochondrial DNA (mtDNA) were observed during myoblast differentiation [8, 9, 16]. Peroxisome proliferator-activated receptor-gamma coactivator 1 alpha (PGC-1α) is a transcription co-activator critically involved in energy metabolism [17, 18]. The PGC-1α is expressed in skeletal muscle and activates mitochondrial biogenesis and oxidative metabolism [17–19].

Tid1, a mitochondrial DnaJ co-chaperone protein (DNAJA3), has been implicated in regulating cell differentiation, cell growth, and cell death [20–25]. In addition, it has been shown that Tid1 is required to maintain the steady-state distribution of mitochondrial membrane potential and is associated with the mtDNA [26]. The Tid1 transcripts are expressed in most of the adult tissues including heart, muscle, bone marrow, spleen, lymph node, and thymus [27]. Mice (αMyHC-Tid1^f/f^) with deficiency of Tid1 specific in cardiomyocytes developed dilated cardiomyopathy (DCM) and died before 10 weeks of age [28]. Cardiomyocytes with Tid1 deficiency also display defective respiratory chain and reduced copy number of mitochondrial DNA [28]. In our previous studies, Tid1 knockout attenuates the mitochondrial mass/volume and induces cell apoptosis in mouse embryonic fibroblasts and pre-matured T cells [24, 25]. However, the regulatory role of Tid1 in skeletal muscle homeostasis remains unclear.

In this study we demonstrated that Tid1 is required to maintain the physiological function in muscular development in vitro and in vivo. The significance of this study warrants further understanding of the function of Tid1 in muscular development. The transgenic mice generated in this study will be a valuable tool to elucidate the physiological function of Tid1 during normal muscular homeostasis.

## Methods

### Cell cultivation and induced differentiation of C2C12 cells in vitro

A detailed protocol for cell cultivation and induced differentiation of C2C12 cells (ATCC® CRL-1772™) is as described previously [29]. In brief, C2C12 cells were expanded in growth medium of Dulbecco’s modified Eagle medium (DMEM), 10% fetal bovine serum (FBS), 1% l-glutamine, and 1% penicillin-streptomycin amphotericin (PSA) at 37 °C under 5% CO_2._ When cultured cells reached 90% confluence, the growth medium was replaced with differentiation medium consisting of DMEM, 2% horse serum (#16050-130, Life technologies), and 1% PSA to stimulate myotube formation. The differentiation medium was replaced every other day during induced differentiation.

### Immunoblotting

Protein extracts from C2C12 cells or mice tissues were prepared using RIPA buffer. The protein amount was measured with the protein assay kit (BioRad). The collected protein extracts were boiled in sample buffer, separated by SDS/PAGE, and transferred onto nitrocellulose membrane. The transferred membrane was incubated in blocking buffer (Tris-buffered saline with 0.1% Tween (TBS-T) and 5% nonfat dry milk), and then blotted with primary antibodies. After washing in TBS-T, the blot was incubated with horseradish peroxidase-conjugated secondary antibody, and the signals were visualized by the enhanced chemiluminescence system as described by the manufacturer (Perkin-Elmer, Wellesley, MA, USA). The blot was re-probed with antibody against GAPDH (Chemicon, Temecula, CA, USA) to confirm equal loading of the protein amount.

### Generation of Tid1 floxed mice and mouse genotyping

The following procedures were performed according to the method described by Lo et al. to generate Tid1^f/f^ mice [24]. In brief, the first loxP site was inserted upstream of the promoter region of Tid1 which is near to a transposon sequence, and the second loxP site was inserted into intron between exon 2 and 3 as indicated in Additional file 1. Deletion of the Tid1 gene in Tid1 floxed mice was achieved by crossing Tid1^f/f^ mice with transgenic Cre mice. After the Cre-mediated recombination, the exon 1 and exon 2 of the Tid1 gene were deleted and the rest of the exon sequences lead to encoding an early stop codon. This truncated protein caused by frameshift mutation contains 18 amino acids whose amino acid sequence is not identical to that of the Tid1 protein. All genotyping of the Cre transgene and the Tid1-deficient mice was performed by polymerase chain reaction (PCR) using genomic DNA isolated from the tail tip. Genomic DNA collected from mice tails was prepared by sodium hydroxide lyses [30]. The genotype screening was confirmed by PCR (the primer sets: IJ-FD-2.1 (5’-GTTTAAGGCCAGTTTGTCTCAAAAC-3’) and KL-RV-3 (5’-ACTTGACTAGCCCTTTAGCATC-3’)).

### Mouse handling and muscular tissue collection

The crossbreeding of homozygous Tid1^flox/flox^ mice (control) with HSA–Cre transgenic mice of a C57BL/6 J background was attempted to give rise to muscle-specific Tid1-deficient mice (HSA-Tid1^f/f^). After birth, mice were weighed and recorded daily. They were then sacrificed at postnatal day (P)5 or P8, and the tissue samples were harvested for morphological, biochemical, and functional analyses.

### Histological and histochemical studies

The collected tissues were dissected, fixed in a 20% formalin neutral buffer solution (Wako, Osaka, Japan) overnight, embedded in paraffin, and sectioned on a microtome using standard techniques. Hematoxylin and eosin (H&E) staining was performed as described previously [31]. For immunochemistry, tissues were incubated with the primary antibodies diluted in TBS-T solution, and then with secondary antibodies. The cross-sectional area was calculated by Image J [32].

### Behavioral experiments

A Rotarod treadmill (Singa Technology Corporation) was used to analyze the motor coordination, balance, and exhaustion resistance. In brief, the mice were placed on a rotating rod running at different speeds and for different durations. The sensor on the bottom of the apparatus recorded the duration of mice falling down from the rotating rod. For data collection, mice were pre-trained for 3 days and trained four times per day on three consecutive days. For each training, the treadmill was held for 90 s with a steady speed of 12 rpm. Subsequently, there were three conditions for trials: test 1 (T1), 90-s trial at a steady speed of 33 rpm; test 2 (T2), 90-s trial at a steady speed of 40 rpm; and test 3 (T3), 600-s trial at a steady speed of 15 rpm.

### Terminal dUTP nick-end labeling (TUNEL) assay

The collected tissues were dissected and embedded within OCT. Next, 7-μm thick frozen sections from mice tissues were sliced in a freezing cryostat at –20 °C. A fluorescein apoptosis detection kit (S7110; EMD Millipore Corporation) was used to measure the apoptotic cells according to the manufacturer's protocol.

### Mitochondrial morphology and membrane potential

The culture medium was removed from the dish and replaced with pre-warmed serum-free culture medium containing 100 nM Mito Tracker Red or 2 μg/mL JC-1. After incubation for 20 min at 37 °C, cells were immediately washed twice in cold phosphate-buffered saline (PBS) and analyzed using a Laser confocal microscope (Olympus FV1000).

### ATP content assay

An ATP measurement kit for cells (K791-100; BIOVISION, USA) was used to measure the ATP concentration according to the manufacturer’s protocol. The ATP concentration was measured using the chemiluminescence produced by the luciferase/luciferin reaction.

### Statistical analysis

Data are expressed as means ± SD. Differences among groups were tested by analysis of variance (ANOVA). Comparisons between two groups were performed by the paired Student *t* test. *P* values less than 0.05 were considered significant.

## Results

### Upregulation of Tid1 during induced differentiation of C2C12 myoblasts

**Fig. 1:**
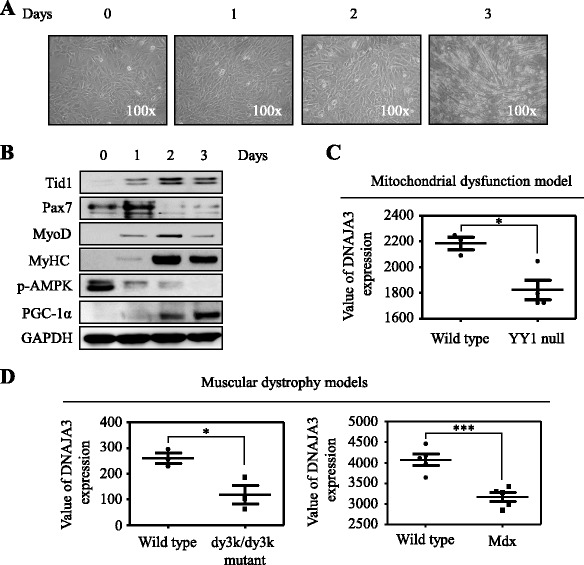
Upregulation of Tid1 during induced differentiation of C2C12 myoblasts. **a** Mouse C2C12 cells were induced to differentiate into myotubes using differentiation medium (DMEM + 2% horse serum) from day 0 to day 3. **b** The crude cell extracts of the C2C12 cells undergoing induced differentiation were prepared and the expression level of proteins of interest was detected by immunoblot analyses. The blot was probed with antibody against GAPDH to confirm equal loading of the protein amount. The expression profile of Tid1 (*DNAJA3*) mRNA was analyzed by the GEO database of the NCBI (http://www.ncbi.nlm.nih.gov/geoprofiles/). Downregulation of Tid1 in the mitochondrial dysfunction (**c**) and muscular dystrophy mice model (**d**). *P < 0.05, ***P < 0.001

To evaluate the expression profile of Tid1 protein during myogenesis, we established and examined mouse myoblast C2C12 cells undergoing induced differentiation into myotubes (Fig. 1a). As shown in Fig. 1b, expression of myogenesis-related markers (myosin heavy chain (MyHC)) was upregulated, whereas PAX7 and MyoD were downregulated during the process of C2C12 differentiation. Of note, the protein level of Tid1 was also increased along with the induced differentiation (Fig. 1b). The decrease of the ATP sensor (p-AMPK) and increase of the mitochondrial biogenesis protein (PGC-1α) indicated that the mitochondrial activity and ATP production were both increased during C2C12 differentiation (Fig. 1b). The GEO Profiles database reveals that Tid1 expression of muscle is downregulated in the mitochondrial dysfunction mice in comparison with those from control mice (GEO accession: GDS4856 [33]) (Fig. 1c). Moreover, the search of the GEO Profiles database reveals that Tid1 expression of muscle is downregulated in muscular dystrophy model mice in comparison with those from control mice (GEO accession: GDS3371 [34] and GEO accession: GDS236 [35]) (Fig. 1d). These results suggest that Tid1 may play a role during myogenesis.

### Phenotypic profile of postnatal transgenic and control mice on day 5

**Fig. 2:**
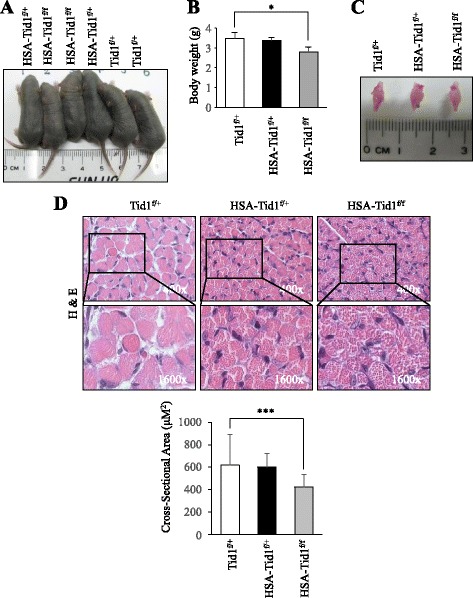
Phenotypic profile of postnatal transgenic and control mice on day 5. **a** Photography of the 5-day-old Tid1^f/+^, HSA-Tid1^f/f^, and HSA-Tid1^f/+^ mice from the same litter, and (**b**) the body weight of the mice was recorded (*n* = 4). **c** Image of the hind limb of mice. **d** Hematoxylin and eosin (*H&E*) staining in the TA muscle of Tid1^f/+^, HSA-Tid1^f/f^, and HSA-Tid1^f/+^ mice. *P < 0.05, ***P < 0.001

To extend the research on understanding the physiological function of Tid1 during muscular development, transgenic mice with muscle-specific Tid1 deficiency (HSA-Tid1^f/f^), for which cre recombinase-mediated deletion of the *Tid1* gene is driven by actin promoter in muscle cells, were generated [36, 37] (Additional file 1). Interestingly, we found that the 5-day-old HSA-Tid1^f/f^ mutant mice displayed less body weight compared to control mice (Fig. 2a and b). However, there was no significant size difference in the hind limb of HSA-Tid1^f/f^ mice in comparison to that of the control mice (Fig. 2c). Next, the histology of the tibialis anterior (TA) muscle was analyzed; as shown in Fig. 2d, the size of muscle fibers of HSA-Tid1^f/f^ mice were reduced compared to those of the control or HSA-Tid1^f/+^ mice.

### More dystrophic phenotype in postnatal HSA-Tid1^f/f^ mice on postnatal day 8

**Fig. 3:**
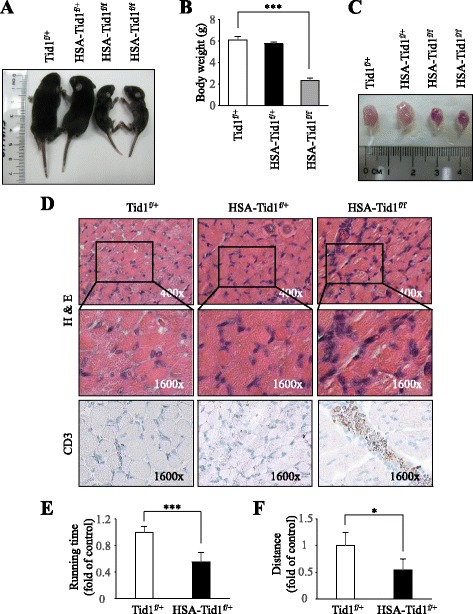
More dystrophic phenotype in postnatal HSA-Tid1^f/f^ mice on day 8. **a** Photography of the 8-day-old Tid1^f/+^, HSA-Tid1^f/f^, and HSA-Tid1^f/+^ mouse from the same litter, and (**b**) the body weight of the mice was recorded (*n* = 3). **c** Image of the hind limb of mice. **d** Hematoxylin and eosin (*H&E*) staining and the CD3 expression stained by immunohistochemistry in the TA muscle of Tid1^f/+^, HSA-Tid1^f/f^, and HSA-Tid1^f/+^ mice. The motor coordination of 6- to 8-week-old Tid1^f/+^ (*n* = 6) and HSA-Tid1^f/+^ (*n* = 3) mice was examined by Rotarod test. The time of running (**e**) and running distance (**f**) of mice were recorded. *P < 0.05, ***P < 0.001

Phenotypically, the HSA-Tid1^f/f^ mice displayed a significant reduction in body weight compared to the control mice on day P8 (Fig. 3a and b), and the size of the hind limb of HSA-Tid1^f/f^ mice was also reduced in comparison to those of either control or HSA-Tid1^f/+^ mice (Fig. 3c). Histologically, the muscle biopsy displayed patchy lymphocytic infiltration in HSA-Tid1^f/f^ mice. The presence of infiltrating lymphocytes indicated the induced inflammation in the muscular tissues of mutant mice (Fig. 3d). In addition, we observed that the HSA-Tid1^f/f^ mice died between days P8 and P10. Because the mice died between P8 and P10, examination of the muscular physiology of adult mice with Tid1 deficiency was prevented. Nevertheless, we evaluated the muscular physiology of adult mice carrying Tid1 heterogeneity (HSA-Tid1^f/+^). We assessed the motor coordination of HSA-Tid1^f/+^ transgenic mice in comparison to that of the control mice. As shown in Fig. 3e and f, we found that the HSA-Tid1^f/+^ transgenic mice displayed reduced running time and running distance. These results suggest that deletion of the Tid1 gene causes dysfunction of muscle tissue of transgenic mice. In addition, Tid1 haploinsufficiency could abolish the physiological muscular function.

### Imbalanced energy and induced apoptosis of muscle from HSA-Tid1^f/f^ mice

**Fig. 4:**
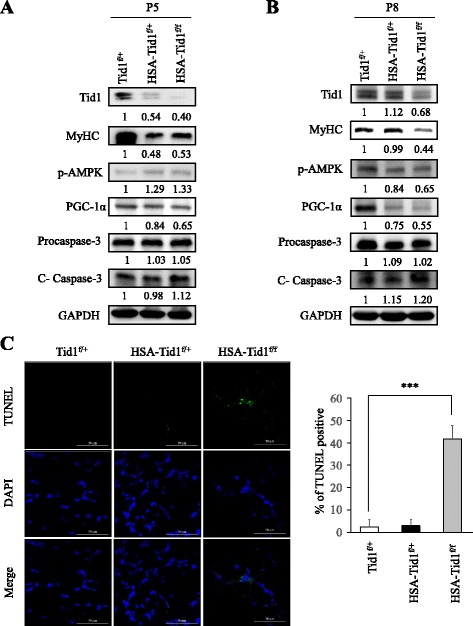
Imbalanced energy and induced apoptosis of muscle from HSA-Tid1^f/f^ mice. Crude proteins extracted from the whole posterior limb of mutant and control mice on day 5 (*P5*) (**a**) and on day 8 (*P8*) (**b**) were collected, respectively. Expression of proteins of interest was detected by immunoblot analyses. **c** The apoptotic cells were detected by TdT-mediated dUTP nick end labeling assay (TUNEL). ***P < 0.001

In order to further investigate the molecular mechanisms involved in muscular homeostasis at different time points, we harvested the protein extracts of muscle tissue from control and mutant mice at P5 and P8. Immunoblotting analyses showed that the expression levels of Tid1 and MyHC proteins were both reduced in HSA-Tid1^f/f^ mice in comparison with those in control mice on P5 and P8 (Fig. 4a and b). In addition, we found that the expression of active AMPK (phosphorylated AMPK) protein within the muscle tissues from HSA-Tid1^f/f^ mice was increased on P5 but reduced on P8 compared to control mice (Fig. 4a and b). Moreover, expression of mitochondrial biogenesis protein (PGC-1α) was also decreased in HSA-Tid1^f/f^ mice (Fig. 4a and b). Of note, we observed that the expression of apoptosis indicator (cleavage Caspase-3) was increased on P5 and P8 in HSA-Tid1^f/f^ mice (Fig. 4a and b). Meanwhile, the Tid1 deficiency induced the number of apoptotic cells within the muscle tissues from HSA-Tid1^f/f^ compared to control mice (Fig. 4c). The above findings suggest that Tid1 deficiency dysregulates energy balance and induces muscular apoptosis during muscular homeostasis.

### Downregulation of Tid1 abolishes the induced differentiation ability of C2C12 by reducing ATP production and impairing mitochondrial activity

**Fig. 5:**
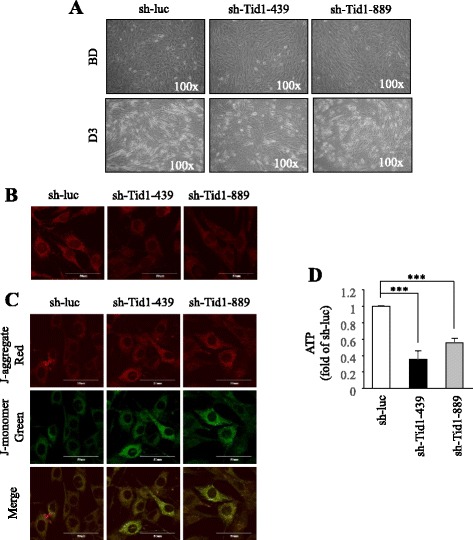
Downregulation of Tid1 impairing mitochondrial activity and ATP concentration. **a** Mouse C2C12 cells were first infected with lentiviral virus (Control: sh-luc; shRNAi-Tid1: sh-Tid1-439 and sh-Tid1-889) to downregulate the Tid1 expression, and the images were acquired before the induced differentiation (*BD*) and on day 3 after the induced differentiation (*D3*). **b**, **c** Confocal images of Mito Tracker staining (**b**) and JC-1 staining (**c**) (indication of mitochondrial membrane potential) of the control and Tid1-knockdown C2C12 before induced differentiation were collected. **d** The intracellular ATP concentration of control and Tid1-knockdown C2C12 before the induced differentiation were measured. ***P < 0.001

**Fig. 6:**
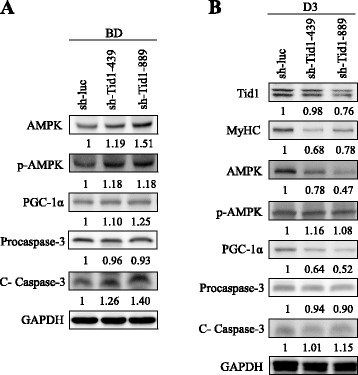
Downregulation of Tid1 inducing apoptosis. The crude cell extracts were prepared from C2C12 cells before induced differentiation (*BD*) (**a**) and on day 3 after the induced differentiation (*D3*) (**b**), and the expression of proteins of interest was detected by immunoblot analyses

To determine whether Tid1 deletion gives rise to abnormal myoblast differentiation in vitro, we manipulated the expression of Tid1 in the myoblast cell line (C2C12) undergoing induced differentiation. Knockdown of Tid1 in C2C12 cells by small hairpin RNA interference (shRNAi) reduced the myotube formation from morphological observation (Fig. 5a). In addition, we observed that C2C12 cells with Tid1 knockdown displayed both reduced mitochondrial mass (Fig. 5b and Additional file 2A) and abolished mitochondrial membrane potential (Fig. 5c and Additional file 2B) before induced differentiation in comparison with control C2C12 cells undergoing regular differentiation, but not on day 3. The intracellular ATP concentration of C2C12 cells with Tid1 knockdown was also decreased before induced differentiation but not on day 3 (Fig. 5d and Additional file 2C). Meanwhile, we found that expression of both total and active AMPK was increased in C2C12 cells with shRNAi-Tid1 before induced differentiation, and the upregulation of AMPK was reduced on induced differentiation day 3 (Fig. 6a and b). In agreement with previous results from HSA-Tid1^f/f^ mice in vivo (see Fig. 4a and b), the expression levels of PGC-1α and MyHC protein in C2C12 cells with Tid1 knockdown by shRNAi were decreased on day 3 (Fig. 6b). We also observed that the expression level of Caspase-3 protein was increased before induced differentiation but not on day 3 within the C2C12 cell undergoing induced differentiation plus Tid1 knockdown (Fig. 6a and b). These findings suggest that Tid1deficiency reduces the differentiation ability of myoblast through reducing ATP production and impairing mitochondrial activity.

## Discussion

The physiological function of chaperone and co-chaperone in muscle cell development remains elusive [38, 39]. In order to further understand the role of Tid1, a mitochondrial co-chaperone, on mediating myogenesis, we determined the expression of Tid1 during the induced differentiation of myoblasts (C2C12 cells) in vitro. During the differentiation of C2C12 cells, we found that the protein levels of Tid1, PGC-1α, and MyHC, as well as the mitochondrial activity, were increased during the process of induced myoblast differentiation (Fig. 1b), consistent with previous studies [8–10, 16]. These results suggest that upregulation of Tid1 may play a role during the myoblast differentiation. Indeed, Linnoila et al. have reported that downregulation of Tid1 impairs the function of the neuromuscular junction [40, 41]. Of note, the HSA-Tid1^f/f^ mice displayed muscular dystrophic phenotype at P8 but not P5 (Fig. 3a–c). In addition, the apoptosis signal cleavage Caspase-3 was enhanced in skeletal muscles of HSA-Tid1^f/f^ mice in comparison with the control mice (Fig. 4a–c). It has been shown that the apoptotic process occurs in muscle fibers of muscular dystrophy phenotype [42–44]. Moreover, we found that AMPK activity was increased at P5 but decreased at P8, and expression of PGC-1α protein was decreased at P8 (Fig. 4a and b). These results suggest that Tid1 deficiency impairs the mitochondrial activity, resulting in insufficient ATP production and cell apoptosis. Next, downregulation of Tid1 by RNA interference reduced the myotube formation and expression of MyHC in the induced C2C12 cells (Figs. 5a and 6b). We also assessed markers of mitochondrial mass/volume (Mito Tracker staining) and ΔΨ (JC-1 staining) by fluorescence microscopy. The Tid1 knockdown impaired the mitochondrial mass and membrane potential (Fig. 5b and c) and reduced the ATP amount compared to those before induced differentiation (Fig. 5d). Meanwhile, the mitochondrial biogenesis marker PGC-1α was reduced in Tid1-knockdown C2C12 cells at day 3 after induced differentiation (Fig. 6b). These in vitro results phenotypically correlate with the HSA-Tid1^f/f^ mice in vivo. However, we did not found the induction of C-caspase 3 by downregulation of Tid1 at day 3 (Fig. 6b). It has been reported that Caspase 3 is required for muscle differentiation [45]. We speculate that the absence of C-caspase-3 changes at day 3 was caused by the induction of Caspase 3 activity of controls during the skeletal muscle differentiation. The discrepancy between the expression of C-caspase 3 under the distinct downregulation of Tid1 and Caspase 3 during the in vitro induced differentiation may be caused by the distinct physiological effect after the gene-specific knockdown. For example, downregulation of Caspase 3 may not cause the mitochondrial abnormality or ATP defect. However, in our previous studies, Tid1 knockout attenuates the mitochondrial mass/volume and induces cell apoptosis in mouse embryonic fibroblasts and pre-matured T cells [24, 25]. Together, these results suggest that downregulation of Tid1 would initially decrease ATP production along with increased cell death, followed by reduced mitochondrial biogenesis (reduction of PGC-1α). These in vitro results imply that energy insufficiency of muscle cells mediated by Tid1 gene deletion (HAS-Tid1^f/f^) is likely the reason for apoptotic cell death during the myogenesis and muscular dystrophy phenotype.

## Conclusion

Our results demonstrate that Tid1 plays a pivotal role in myogenesis. Deletion of the Tid1 gene causes dysfunction of muscle tissues of transgenic mice. Downregulation of Tid1 abolished the ability of C2C12 to differentiate via reduced ATP production, impaired mitochondrial activity, and induced apoptotic cell death.

In summary, Tid1 deficiency reduces ATP production, impairs the mitochondrial activity of muscle cells during the myogenesis, and, consequently, causes muscle cell apoptosis. However, how Tid1 deficiency reduces ATP production and inhibits PGC-1α activity remains unclear. Thus, we will further investigate the molecular mechanisms mediated by Tid1 to regulate ATP production and PGC-1α activity during myogenesis. In addition, our muscle-specific Tid1 deletion mice will provide a useful tool to study the physiological function of Tid1 in normal muscular homeostasis.

## Additional files

Additional file 1: Targeted disruption of Tid1 gene in transgenic mice. (A) Partial genomic maps of the floxed locus after HSA-Cre-mediated Tid1 deletion. The exon 1, exon 2, and exon 3 of Tid1 gene are indicated by the solid box. (B) Genomic DNA extracted from the tail of mutant and control mice (postnatal day 5) was collected and genotyped by PCR analyses. (PDF 64 kb)
Additional file 2: Downregulation of Tid1 during the induced differentiation of C2C12. Confocal images of Mito Tracker staining (A) and JC-1 staining (B) (indicative of mitochondrial membrane potential) of the control and Tid1-knockdown C2C12 on day 3 during the induced differentiation were collected. (C) The intracellular ATP concentration of control and Tid1-knockdown C2C12 on day 3 during the induced differentiation was measured. (PDF 93 kb)

## Abbreviations

ATP: Adenosine triphosphate
DAPI: 4′,6-Diamidino-2-phenylindole
DMEM: Dulbecco’s modified Eagle medium
HSA: Human α-skeletal actin
mtDNA: Mitochondrial DNA
MyHC: Myosin heavy chain
P: Postnatal day
PCR: Polymerase chain reaction
PGC-1α: Peroxisome proliferator-activated receptor-gamma coactivator 1 alpha
PSA: Penicillin-streptomycin amphotericin
