# Supplementary material for: Mitochondrial co-chaperone protein Tid1 is required for energy homeostasis during skeletal myogenesis
Source: Stem Cell Res Ther. 2016 Dec 7;7:185. doi: 10.1186/s13287-016-0443-8 (PMC5143475; doi:10.1186/s13287-016-0443-8)
Supplement: Additional file 1: — Targeted disruption of Tid1 gene in transgenic mice. (A) Partial genomic maps of the floxed locus after HSA-Cre-mediated Tid1 deletion. The exon 1, exon 2, and exon 3 of Tid1 gene are indicated by the solid box. (B) Genomic DNA extracted from the tail of mutant and control mice (postnatal day 5) was collected and genotyped by PCR analyses. (PDF 64 kb) [file 13287_2016_443_MOESM1_ESM.pdf]

Additional file 1, Cheng et al.

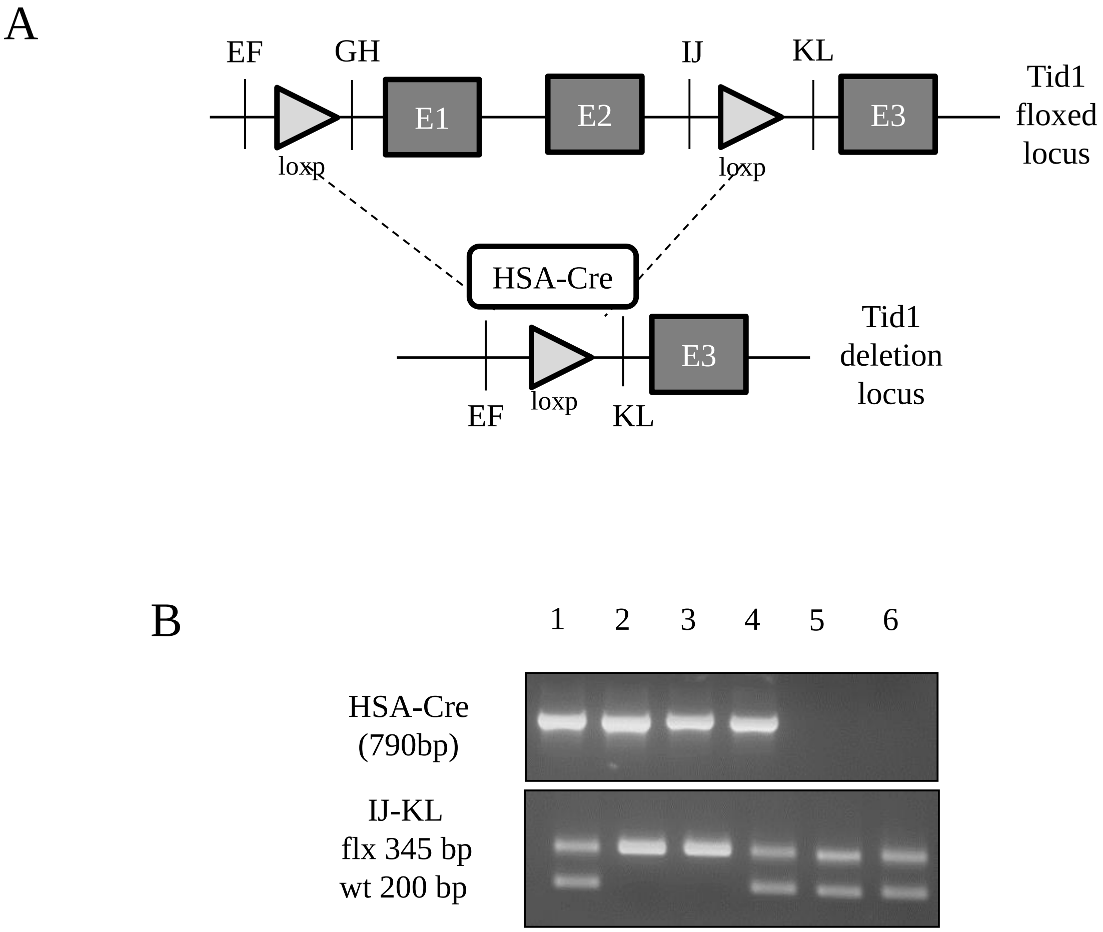

Additional file 1. Targeted disruption of Tid1 gene in transgenic mice.
(A) Partial genomic maps of the floxed locus after HSA-Cre-mediated Tid1 deletion. The exon 1, exon 2 and exon 3 of Tid1 gene are indicated by solid box. **(B)** Genomic DNA extracted from the tail of mutant and control mice (postnatal day 5) was collected and genotyped by PCR analyses.
