# Supplementary material for: Mitochondrial co-chaperone protein Tid1 is required for energy homeostasis during skeletal myogenesis
Source: Stem Cell Res Ther. 2016 Dec 7;7:185. doi: 10.1186/s13287-016-0443-8 (PMC5143475; doi:10.1186/s13287-016-0443-8)
Supplement: Additional file 2: — Downregulation of Tid1 during the induced differentiation of C2C12. Confocal images of Mito Tracker staining (A) and JC-1 staining (B) (indicative of mitochondrial membrane potential) of the control and Tid1-knockdown C2C12 on day 3 during the induced differentiation were collected. (C) The intracellular ATP concentration of control and Tid1-knockdown C2C12 on day 3 during the induced differentiation was measured. (PDF 93 kb) [file 13287_2016_443_MOESM2_ESM.pdf]

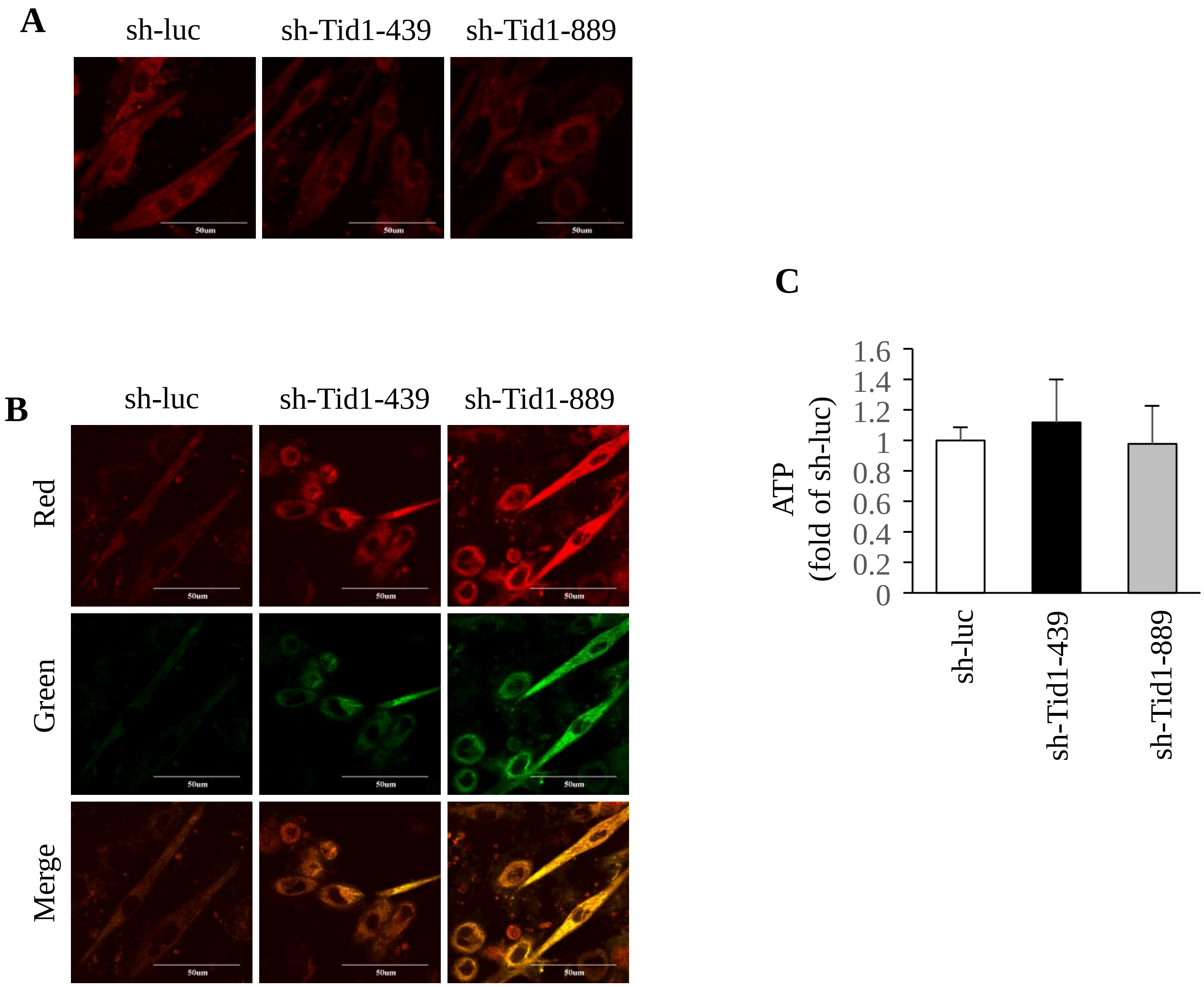

Additional file 2. Down-regulation of Tid1 during the induced differentiation of C2C12.

(A) Confocal images of Mito Tracker staining (A) and JC-1 staining (B) (indication of mitochondrial membrane potential) of the control and Tid1-knockdown C2C12 on day 3 during the induced differentiation were collected. **(C)** The intracellular ATP concentration of control and Tid1-knockdown C2C12 on day 3 during the induced differentiation was collected.
